# Phage Display Approaches for the Isolation of Monoclonal Antibodies Against Dengue Virus Envelope Domain III from Human and Mouse Derived Libraries

**DOI:** 10.3390/ijms13032618

**Published:** 2012-02-27

**Authors:** Nicole J. Moreland, Patricia Susanto, Elfin Lim, Moon Y. F. Tay, Ravikumar Rajamanonmani, Brendon J. Hanson, Subhash G. Vasudevan

**Affiliations:** 1 Duke-NUS Graduate Medical School, 8 College rd, Singapore; E-Mails: n.moreland@duke-nus.edu.sg (N.J.M); yuefeng.tay@nus.edu.sg (M.Y.F.T); 2 Defence Medical and Environmental Research Institute, DSO National Laboratories, Singapore; E-Mail: hbrendon@dso.org.sg

**Keywords:** dengue, E protein, domain III, phage display, Fab, hybridoma

## Abstract

Domain III of the dengue virus envelope protein (EDIII, aa295-395) has an immunoglobulin fold and is the proposed receptor-binding domain of the virus. Previous studies have shown that monoclonal antibodies against EDIII can be neutralizing and have therapeutic potential. Here, cloned Fab-phage libraries of human and mouse origin were screened for DENV specific antibodies. Firstly, bacterially expressed EDIII or whole virus particles were used as bait in biopanning against a large naïve human Fab-phage library (>10 billion independent clones). Multiple panning strategies were employed, and in excess of 1000 clones were screened, but all of the antibodies identified bound the envelope in regions outside EDIII suggesting EDIII antibodies are virtually absent from the naïve human repertoire. Next, a chimeric Fab-phage library was constructed from a panel of EDIII specific mouse hybridomas by pooling the VH and VL chain sequences from the hybridomas and cloning these into the pComb3X phagemid vector with human CH and CL encoding sequences. Biopanning against EDIII identified a unique antibody (C9) that cross-reacts with EDIII from DENV1-3 and, in the IgG format, binds and neutralizes DENV2 in cell-based assays. Sequence analysis and saturation mutagenesis of complementary determining regions (CDR) in the C9 light chain suggest an antigen recognition model in which the LCDR3 is a key determinant of EDIII specificity, while modifications in LCDR1 and LCDR2 affect DENV serotype cross-reactivity. Overall, this study supports the current prevailing opinion that neutralizing anti-EDIII monoclonal antibodies can be readily generated in murine systems, but in humans the anti-DENV immune response is directed away from domain III.

## 1. Introduction

Dengue virus (DENV) is a mosquito-borne *Flavivirus* responsible for at least 100 million symptomatic infections each year. There are four circulating serotypes of dengue (DENV1-4) that show approximately 70% sequence homology [1]. A primary infection provides effective long-term protection against re-infection with the same serotype, but can increase disease severity upon infection with a different serotype. This process, termed antibody dependent enhancement (ADE), is thought to be facilitated by poorly neutralizing cross-reactive antibodies generated in the primary infection that promote virus entry via Fcγ receptor bearing cells such as monocytes [2,3].

The envelope (E) protein of DENV is the major target of the humoral response following a DENV infection, although B-cell responses towards the structural protein, precursor membrane (prM), and non-structural protein 1 (NS1) have also been reported [4–6]. Structural analysis of the flavivirus E protein has identified three beta barrel domains: EDI, EDII and EDIII, with the native protein forming a head-to-tail homodimer [7]. Domain II has a long, finger-like structure and harbors the fusion peptide, while EDIII has an immunoglobulin-like fold and is the proposed receptor-binding domain of the virus [8–10]. EDI connects domain II and III to form the hinge region and plays a pivotal role in the structural rearrangement of E from a homodimer to a trimer, which occurs on exposure to acidic conditions in the trans-Golgi secretory pathway. The reorganized E trimer exposes the hydrophobic fusion loop in EDII to mediate cellular fusion [11].

**Figure 1:**
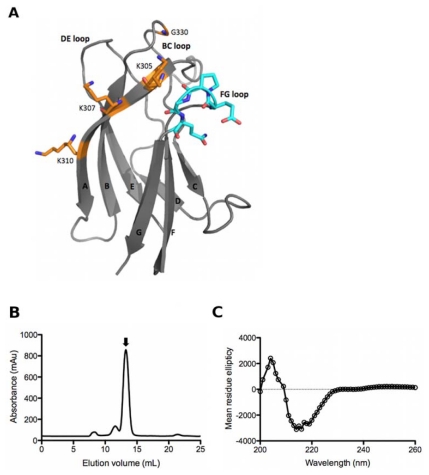
(**A**) Structure of dengue virus 2 (DENV2) E protein domain III (aa295-395) in ribbon representation (PDB 1OAN). Residues comprising the 9F12 epitope are marked in orange including conserved lysines in the A-strand (K305, K307, K310) and G330 on the BC loop. Residues recognized by 3H5 in the FG loop are marked in cyan; (**B**) size exclusion chromatography (SEC) profile for refolded DENV1 EDIII protein using a S75 10/300 column. The major elution peak corresponds to monomeric EDIII (marked by arrow); (**C**) Far-UV circular dichroism spectra of DENV3 EDIII.

Murine monoclonal antibodies (mAbs) that bind all three domains of E have been identified, but the most potent neutralizing murine mAbs tend to bind to EDIII [12–15]. Epitope mapping studies have shown that murine mAbs with the strongest neutralizing activity are generally serotype specific and bind the lateral ridge region of EDIII (BC, DE and FG loops, Figure 1) where sequence diversity between serotypes is high, while cross-reactive murine mAbs that bind conserved lysines on the A strand (Figure 1) are generally weaker neutralizers [12,15]. Until very recently the immune repertoire generated in response to DENV infections in humans was poorly characterized, but an emerging body of data suggests that the strong immune response to EDIII exhibited by mice may not be mirrored in humans. Serological studies have found the relative proportion of EDI/EDII reactive antibodies generated following DENV infections in humans is significantly higher than EDIII reactive antibodies [16,17], and in two separate analyses the anti-EDIII response in human sera has been shown to contribute little or nothing to overall DENV neutralization [16,18]. However, human EDIII-specific mAbs generated by B-cell immortalization were shown to neutralize DENV in a lethal mouse model [19], suggesting that EDIII specific antibodies, though individually neutralizing, are of low abundance in the human repertoire.

In this study cloned antibody libraries of human and mouse origin have been utilized, as sources of DENV specific antibodies, in a bid to further understand the anti EDIII immune response. Initially a large (>10 billion independent clones), naïve Fab (fragment antibody binding) phage library derived from 14 non-immune human donors was panned using EDIII or whole virus particles as bait, and several DENV specific Fab were isolated and evaluated for their binding specificities. In an alternative approach, a Fab-phage library was constructed from a panel of EDIII specific mouse hybridomas. The hybridomas had been obtained by immunizing BALB/c mice with EDIII from DENV2, and included 9F12, which has been shown to neutralize DENV in a prior study [20]. Using phage display to clone antibodies from hybridomas enables contaminating, non-functional, heavy and light chain (VH and VL) mRNA sequences often present in hybridomas to be eliminated during biopanning [21]. Biopanning against EDIII identified unique clones with specificity for DENV, the most promising of which cross-reacts with EDIII from DENV1-3.

## 2. Results

### 2.1. Biopanning the Naïve Human Fab Library (HX02) with EDIII

EDIII proteins from all four DENV serotypes were purified and refolded with yields ranging 2–10 mg/L of culture. Correct folding of the proteins was confirmed by size exclusion chromatography (SEC), which showed each of the EDIII proteins to be monomeric, and circular dichroism (Figure 1). The circular dichroism spectrum for each of the proteins had a nadir at 218 nm indicating the secondary structure was predominantly β-sheet in agreement with the published structures for DENV EDIII (Figure 1, [7]).

For EDIII biopanning with HX02, three different biopanning strategies were employed and varying levels of stringency applied in repeated attempts to enrich for EDIII binding clones (Table 1). Initially EDIII from DENV1 was coated onto an immunotube at a concentration of 50 μg/mL and subject to three rounds of biopanning using *E. coli* TG1 cells to amplify phage. Next, EDIII from DENV1 was coated into an immunoplate with a starting concentration of 20 μg/mL and *E. coli* XL-I blue cells were used to amplify phage in six rounds of biopanning. Finally, EDIII from DENV1 and 2 was biotinylated and immobilized on magnetic streptavidin resin for solution based panning. Such solution-based approaches avoid denaturation of epitopes that can occur when coating antigens in plastic [22]. Despite screening in excess of 1000 clones across these three different strategies by phage enzyme-linked immunosorbant assay (ELISA), no EDIII specific antibodies were identified from the HX02 naïve human library when the EDIII domain was used as bait.

### 2.2. Biopanning the HX02 Library with DENV Virus Particles

**Figure 2:**
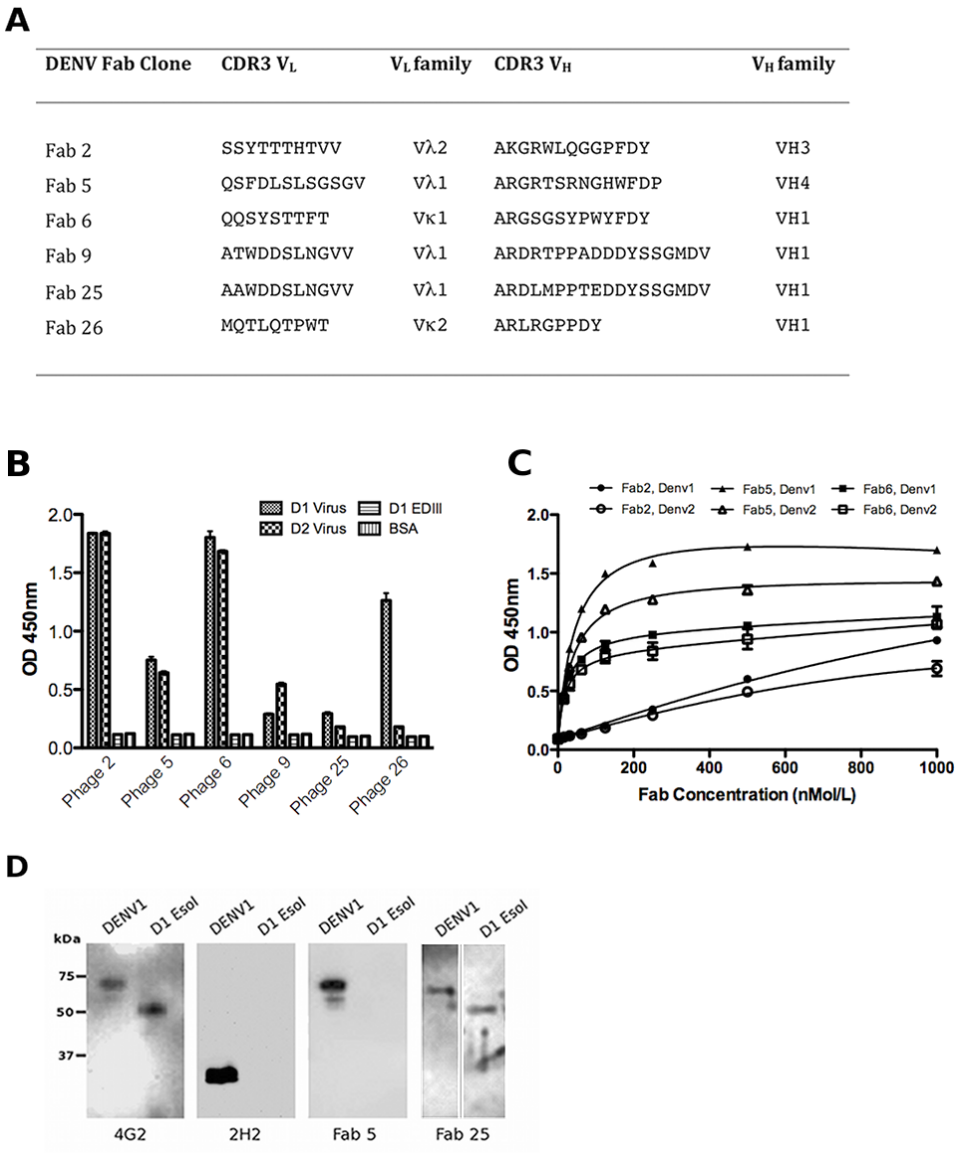
Biopanning the HX02 naïve library with DENV1 whole virus particles and characterization of the anti-DENV Fabs identified. (**A**) Sequence analysis and variable gene usage of anti-DENV Fab. Shown are the complementary determining region 3 (CDR3) and variable gene family designations, assigned using IMGT/V Quest [24] (**B**) Phage ELISA of the six unique Fab-phage clones against DENV1 virus (small check), DENV2 virus (large check), DENV1 EDIII (horizontal line) and BSA (vertical line); (**C**) An ELISA of purified Fab against DENV1 virus (closed symbols) and DENV2 virus (open symbols); (**D**) A Western blot of selected Fab with whole virus and Esol from DENV1. 4G2 and 2H2 are control mouse mAbs, which bind E and prM proteins, respectively.

To determine the presence of anti-DENV envelope antibodies in the naïve library, DENV1 virus particles were purified from the supernatant of infected C6/36 cells using a sucrose gradient and used as antigen. Four rounds of panning against DENV1 virus coated onto an immunoplate at 20 μg/mL were performed with HX02 phage (Table 1). Success was evident by a primary screen of 384 clones by phage ELISA with DENV1 that gave 188 positive hits. Diagnostic *Bst*N1 digests enabled grouping of clones with similar DNA fingerprints and sequencing confirmed the identification of six unique clones. Sequence analysis with IMGT/V-QUEST showed that the VH sequences belong to the VH1, VH3 or VH4 gene families while the variable light VL sequences selected are derived from both kappa and lambda gene families (Vκ1, Vκ2, Vλ1, Vλ2) (Figure 2A). The heavy chain CDR3 sequences, which have a large influence over antibody binding specificity [23], are diverse in length and composition.

To ascertain the specificity and DENV cross-reactivity of the six unique Fab phage clones a phage ELISA was performed against DENV virus particles from serotypes 1 and 2 and EDIII from DENV1. All of the six Fab-phage clones gave clear positive reactivity with the biopanning antigen, DENV1 virus, compared with the control BSA (Figure 2B). Fab-phage clones 2, 5, 6 and 9 also gave clear signals against DENV2 virus suggesting these clones are cross-reactive. None of the clones had reactivity with EDIII indicating the epitopes for these clones are not contained within domain III (aa295-395) of the viral envelope protein.

### 2.3. Characterisation of the Unique Anti-DENV Antibodies

The phagemids of the six unique clones were digested with *Sal*I to remove the gene III sequence and enable expression of the soluble Fab in *E. coli* Top10 F′ cells. ELISA with the six purified Fabs confirmed binding to DENV virus as shown for Fab 2, 5 and 6 in Figure 2C. Fab 5 had the highest reactivity with DENV1 and DENV2 of the group of Fabs. As with the phage ELISA, none of the purified Fabs had reactivity with EDIII (data not shown). In the absence of any EDIII binders, DENV virions were resolved by SDS-PAGE and subjected to Western blot to identify the viral structural protein recognized by the Fabs. All of the Fab, and the 4G2 control that binds the fusion loop in EDII, detected a protein of approximately 65 kDa (Figure 2D), which corresponds to the viral envelope protein. None of the Fabs detected the 25 kDa protein that corresponds to precursor membrane (prM) protein seen with the 2H2 control. Intriguingly, four of the Fab (2, 5, 6 and 26) bound whole virus particles, but not the recombinantly expressed, soluble Ectodomain (Esol, aa1-400). The remaining Fab (9 and 25) detected Esol by Western blot, and since they are negative against EDIII (Figure 2A), it can be assumed their epitopes are contained within aa1-290 of Esol (EDI and EDII).

As Fab 5 bound DENV with the highest avidity (Figure 2C), the ability of the antibody to neutralize DENV1 virus was examined. It was only weakly neutralising in the Fab format (30% neutralization at 1.75 mg/mL) and as such this panel of human Fabs was not pursued further.

### 2.4. Generation and Biopanning with the Chimeric Library

To generate a phage library highly enriched for EDIII binding Fab, the mRNA from 10 hybridomas previously shown to bind DENV2 EDIII [20] were collected. The Vκ and VH encoding sequences from each hybridoma were separately amplified using published protocols [25], and then pooled in equal quantities to facilitate chain swapping during library generation and the generation of novel Fab in the enriched anti-EDIII hybridoma library. The Vκ and VH pools were subject to overlap PCR to generate Vκ/human Cκ and mouse VH/human CH cassettes, which were ligated into the pComb3X phagemid. The resulting chimeric library comprised approximately 2 × 10^6^ independent transformants. Phage ELISAs with clones from the transformation plate showed 5/40 (12.5%) of clones were positive for DENV2 EDIII before biopanning. Over 90% of clones sequenced from the unselected library encoded functional Fab and there were several Fab with the same heavy chain and different light chain (and vice versa) indicating chain swapping had occurred during library generation.

**Figure 3:**
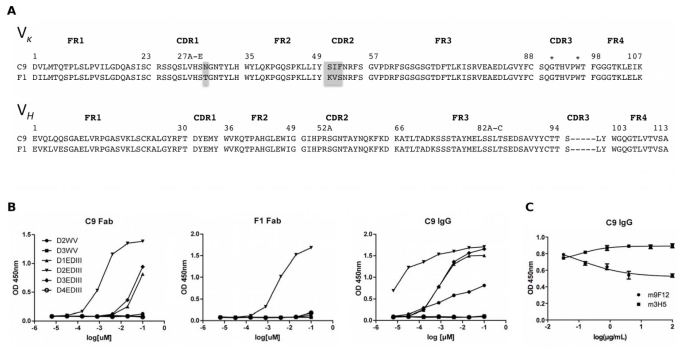
(**A**) Amino acid sequence alignment of mouse variable domains of chimeric mouse/human Fab clones C9 and F1. Shown are the framework regions (FR) and complementary determining regions (CDR) of the Vκ1 and VH1 using Kabat numbering [26]. Notable sequences differences are highlighted in grey and G91 and W96 in LCDR3 are marked (*); (**B**) Binding of C9 and F1 Fab and C9 IgG to DENV1-4 EDIII and DENV2 and 3 whole virus (D2WV, D3WV); (**C**) Elisa for C9 IgG (30 nmol/L) against DENV2 whole virus particles competed with increasing concentrations of mouse 9F12 and 3H5 IgG. C9 IgG binding was detected with an anti-human horseradish peroxidase (HRP) conjugate.

Three rounds of panning with DENV2 EDIII coated on an immunoplate were performed with the chimeric library. In a phage ELISA 44/96 (45.8%) of clones were positive for EDIII demonstrating selection had occurred during biopanning. Selected clones were subject to sequencing and it was found that all positive clones had one of two Fab sequences, C9 or F1 (Figure 3A). These Fab-phage clones express the same heavy chain (VH1), and their light chain framework (Vκ1) and CDR3 are identical, but they differ in the CDR1 and CDR2 in their light chain.

### 2.5. Characterisation of Anti-EDIII Fab and IgG

The phagemids for C9 and F1 were transformed into *E. coli* Top10 F′ cells for Fab expression and the specificity and DENV cross-reactivity of the Fabs were assessed by ELISA (Figure 3B). C9 had strong binding for EDIII from DENV2 and to a lesser extent with EDIII from DENV1 and DENV3. It had no reactivity with EDIII from DENV4. F1 also had strong binding with EDIII from DENV2, but the cross-reactivity with EDIII from DENV1 and 3 was reduced compared with C9. The reactivity with whole virus from DENV2 and DENV3 for both C9 and F1 was low in the Fab format.

Given the superior cross-reactivity profile of C9, this Fab was cloned into the PIGG vector [27] for IgG expression in HEK293T cells. The C9 IgG had the same cross-reactivity profile as C9 Fab with the highest avidity seen with DENV2 EDIII, followed by EDIII from DENV1 and 3. On average, conversion of the monovalent Fab to bivalent IgG resulted in a 30 times stronger interaction with each EDIII antigen, which is comparable to published avidity gains [28]. Despite having reactivity with EDIII from DENV3, the IgG did not detect whole virus particles from DENV3, but binding with DENV2 whole virus is clearly observed in the IgG format (Figure 3B).

To ascertain the binding site for C9 IgG on EDIII, competition ELISAs were performed with two well-characterized EDIII specific mAb (Figure 1B); 3H5 for which the epitope has been shown to include aa383-386 in the FG loop [29], and 9F12 that binds the A-strand and G330 in the BC loop [20]. DENV2 virus was coated onto an immunoplate and incubated with increasing concentrations of mouse 3H5 and 9F12. C9 IgG was added at a fixed concentration of 30 nmol/L and binding was detected with an anti-human IgG HRP conjugate. High concentrations of 9F12, but not 3H5, partially competed with C9 IgG binding (Figure 3C), indicating there is some overlap between the epitopes for 9F12 and C9.

**Figure 4:**
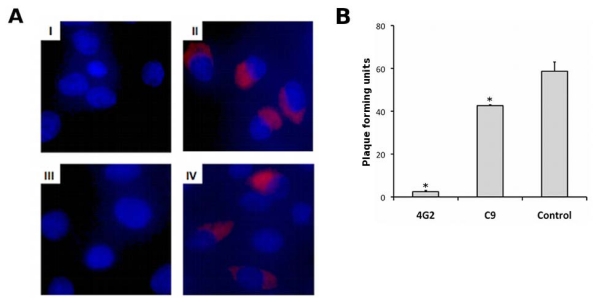
(**A**) Immunofluorescence of uninfected BHK-21 cells (panel I and III) and cells infected with DENV2 (panel II and IV). Cells were incubated with C9 IgG (panel I and II) or 4G2 (3 nmol/L, panel III and IV) followed by goat anti-human or anti-mouse AF594 (Invitrogen), and mounted with ProLong Gold containing DAPI; (**B**) Plaque reduction neutralization test performed with DENV2 in BHK21 cells. Virus was incubated with C9 IgG or 4G2 (1 μM) prior to infection. Statistical significance (*p* < 0.001) is marked (*).

The reactivity of the C9 IgG with the DENV virion was further examined using immunofluorescence staining of DENV2 infected BHK 21 cells (Figure 4A). Both C9 IgG, and the control antibody 4G2, produced clear fluorescence intensity in the cytoplasm of DENV2 infected cells 24 h post infection (panel II and IV), with no evidence of cross reactivity with other cellular components in the uninfected cells (panel I and III). The neutralization of C9 IgG against DENV2 was examined using plaque reduction neutralization tests (PRNT). The purified IgG was moderately neutralizing. At a concentration of 1 μM (0.15 mg/mL) it significantly reduced the number of plaques formed by 25% (*p* < 0.001), compared with a 95% reduction observed with 4G2 (Figure 4B). The 50% neutralization point for C9 IgG is 0.85 mg/mL, compared with 0.015 mg/mL for 4G2, the positive control (data not shown).

### 2.6. Saturation Mutagenesis of C9 Light Chain

In an attempt to improve the affinity and neutralization of C9 a Fab-phage library was constructed to optimize the VL CDR3. This CDR was focused on as, although the VH and VL CDR3 are generally responsible for high affinity interactions with antigen, some studies have shown that mutations in the VH CDR3 almost always abolish binding [30]. Comparison of the C9 light chain sequence with F1 (Figure 3A) also suggested that alterations within the VL CDR1 and CDR2 could reduce the cross reactivity propensity of the Fab.

For VL CDR3 optimization, a segment of 7 amino acids, which covers the V-J joining region was randomized by NNK doping as previously published [25,27]. Randomisation of the G91 and W96 within the CDR3 was avoided (Figure 3A) as glycine is critical for turns and tryptophan often has a critical structural role in CDRs [30,31]. Overlap PCR with the NNK doped primers yielded a library of 4 × 10^5^ independent transformants. Twelve test clones sequenced from the transformation plate had a unique VL CDR3 with the exception of the non-randomized glycine and tryptophan. The LCDR3 Fab-phage library was selected by four rounds of biopanning against DENV2 EDIII immobilized in an immunoplate. A highly stringent off-rate selection was included in rounds 2–4. Phage were allowed to compete for binding to immobilized EDIII in the presence of high concentrations of EDIII for 24 h, as the rate of dissociation often distinguishes primary from affinity matured antibodies [32]. Approximately 70% of clones were positive for EDIII in a phage ELISA following biopanning. However every positive clone sequenced had a LCDR3 identical to C9. Only clones that did not bind EDIII had unique VL CDR3 sequences suggesting that, at least amongst the 4 × 10^5^ clones in the library, the residues in C9 LCDR3 are optimal for antigen recognition.

## 3. Discussion

This study has used phage display as a central technique to identify and characterize anti-DENV antibodies, with the primary goal being the isolation of an EDIII specific antibody, since both human and mouse antibodies that target EDIII have been shown to be highly neutralizing [15,19]. Initial investigations with a naïve human library (HX02) and recombinant EDIII as antigen did not yield any anti-EDIII antibodies despite multiple panning strategies and exhaustive clone screening. This lack of EDIII specific clones is unlikely to be a reflection of library quality, but rather extremely low levels of anti-EDIII antibodies in the naïve human repertoire since DENV positive/EDIII negative Fab were identified when whole virus particles were used as antigen. Furthermore, we and others have successfully used the HX02 library to isolate antibodies specific for dengue non-structural proteins [33,34] and hemagglutinin of H5N1 influenza [35]. Previous phage display studies with the closely related WNV also support the hypothesis. When a naïve human scFv library was used for panning with purified WNV E protein, 11 antibodies with specificity for EDI and EDII were identified, but no antibodies with specificity for EDIII were isolated [36]. EDIII specific antibodies were identified in a subsequent study where immune libraries derived from three WNV infected patients were used for biopanning [37]. But even then, the proportion of EDIII specific mAbs was low (4/51, 8%) compared with domain II (24/51, 47%).

The six Fabs identified by panning the HX02 library against DENV1 virus all bound the DENV envelope, but only two of them reacted with Esol (aa1-400). The Esol used in this study lacks the stem/transmembrane region (aa401-495) of the viral envelope protein and it is possible that some of the Esol negative Fab bind to these regions. Some may also bind E protein epitopes that are only available in the context of the native virion. Similar observations were made in two recent studies that examined the antibody response of humans exposed to primary DENV infections where over 90% of the mAbs generated bound DENV virus particles but not Esol [19,38]. The majority of mAbs identified in these studies were weakly neutralizing, and it was concluded that only a small fraction of DENV-specific antibodies in immune sera are responsible for neutralization. Our neutralization tests with Fab 5, which had the highest reactivity with DENV virus particles by ELISA, were not encouraging suggesting that a non-immune, human repertoire is not an ideal source of neutralizing antibodies for DENV.

To increase the possibility of isolating neutralizing, EDIII specific mAbs by phage display, an immune library could be generated from DENV infected patients. However, the low percentage of EDIII positive clones in WNV immune libraries [37], and the DENV studies with immune sera that show EDIII mAbs are of low abundance in the human repertoire [19,38] suggest biopanning a human, DENV immune library would not be without challenges. As an alternative we decided to exploit the strong anti-DENV EDIII response exhibited by mice [12,13,15], and generated a chimeric mouse human Fab phage library in which the VL and VH were derived from a panel of previously generated anti-EDIII hybridomas [20], and the CL and CH were of human origin. Constructing a chimeric library ensures that the Fabs have a human constant region, which would be beneficial for latter therapeutic development. The two Fabs identified by biopanning against EDIII, C9 and F1, share the same heavy chain suggesting that of the 10 heavy chains included in the initial mRNA pool, this VH1 chain has superior EDIII binding. The Fabs had strong reactivity with DENV2 EDIII, but the cross-reactivity of F1 towards EDIII from DENV1 and 3 was reduced compared with C9. The VL CDR1 and CDR2 in C9 and F1 differ in sequence, and though the Asn to Thr mutation in F1 LCDR1 is relatively conservative, the gain of positive charge in the F1 LCDR2 conferred by the Ser to Lys mutation may affect hydrogen bonding and antigen binding. Saturation mutagenesis of the LCDR3 in C9 indicated that any amino acid substitutions in this CDR abolish EDIII binding. This is consistent with an antigen recognition model in which the highly diverse CDR3 loops are the key determinants of specificity and modifications in the germ-line encoded CDR1 and CDR2 sequences have less impact [23].

Conversion of C9 to an IgG resulted in good avidity gains against DENV1-3 EDIII antigens and DENV2 whole virus, though the IgG had no reactivity with DENV3 despite binding DENV3 EDIII. The C9 epitope overlaps, in part, with that of 9F12 (A strand and BC loop), but a significant portion of the C9 epitope must be DENV2 specific since it has the highest reactivity to DENV2 EDIII. The lack of C9 binding with the DENV3 virion implies the residues that enable C9 to cross-react with DENV1-3 EDIII are not solvent accessible in the DENV3 virion. In effect this means that even with DENV2, not all of the C9 epitope can be engaged in the context of the virion. This probably explains why the IgG is only moderately neutralizing as epitope accessibility has been correlated with neutralization [39]. The moderate neutralization exhibited by C9 IgG is comparable to that of 9F12 IgG [20], but is several orders of magnitude higher than recently discovered anti-EDIII mAbs with therapeutic potential [13,19]. Nevertheless, C9 is a well-characterized antibody and a useful research tool. Its isolation has provided an EDIII specific antibody that can be readily produced without the concern of cell-line instability associated with hybridomas [21], and the human Fc enables antibody dependent enhancement studies to be conducted with human cell lines [18,19].

## 4. Materials and Methods

### 4.1. Antigen Preparation

EDIII antigens (aa295-395) from each of the four serotypes (DENV1 Hawaii, Genbank X76219; DENV2 TSV01, AY037116; DENV3 H87, M93130; DENV4 H241, AY947539) were refolded from inclusion bodies following expression in *E. coli* BL21 (DE3) cells as described [20] with minor modifications. Briefly, inclusion bodies were washed in 1 M urea and 2% Triton X 100, clarified by centrifugation, and solubilized in 20 mM Tris-HCl pH 8.5, 8 M urea, 150 mM NaCl, 10 mM imidazole and 10 mM β-ME. Proteins were loaded onto a Ni-NTA column (GE Healthcare) pre-equilibrated with the same buffer, eluted with an imidazole gradient and collected for refolding. Refolding was performed by dialysis in 200 mM Tris pH 8.5, 10 mM EDTA, 5 mM reduced glutathione, 0.5 mM oxidative glutathione and 100 mM arginine. A final SEC step with a Superdex 200 10/300 column (GE Healthcare) was conducted in 20 mM Tris pH 8.5 and 250 mM NaCl prior to storage.

For whole virus particles, mosquito C6/36 cells were infected with DENV1-4 (serotypes as above) at an MOI of 0.1. The infected cell supernatant was harvested on day 5 post-infection and layered on top of a sucrose gradient ranging 11–55% in Phosphate Buffered Saline (PBS). Following centrifugation at 4 °C, 75,000 *g* for 18 h, fractions containing DENV were pooled, concentrated and buffer exchanged into PBS using a centrifugal concentrator (MWCO 100,000 Da, Millipore). Infectious titer was determined by a standard plaque assay on BHK 21 cells. Virus particles were UV-inactivated prior to use in biopanning experiments, and quantified by Western blot with 4G2.

The recombinant ectodomain of DENV1 (Esol, aa1-400) was a kind gift from Drs Petra Kukkaro and Shee Mei Lok (Duke-NUS Graduate Medical School, Singapore). The protein was expressed in *Drosophila melanogaster* S2 cells and purified as previously described [7].

### 4.2. HX02 Fab-Phage Library Biopanning

Library screening was performed with a naïve human Fab phage display library HX02 (Humanyx Pte Ltd, Singapore) displayed in a modified pCES1 vector [40]. Panning was performed as described previously [33] but several different antigen immobilization strategies were utilized (Table 1). In all experiments the number of PBS-T (0.1% Tween-20) wash steps was increased, and antigen concentration decreased, during the biopanning cycles to increase stringency. Initially, EDIII from DENV1 was coated onto an immunotube (Nunc International) at a concentration of 50 μg/mL in PBS, pH 7.4. Phage (1 × 10^12^) were added to the tube and allowed to bind for 1.5 h at room temperature. Non-specifically bound phage was removed with the number of wash steps shown in Table 1 and bound phage was eluted with 100 mM triethylamine. Eluted phage was used to infect *E. coli* TGI cells, cultures were rescued with M13K07 helper phage, and amplified phage was concentrated by polyethylene glycol-NaCl precipitation

In the next panning experiment, EDIII from DENV1 was coated in four wells of an immunoplate (Nunc International) at a concentration of 10 μg/mL in 50 mM Na_2_CO_3_ pH 9.6. Phage were added to the wells and allowed to bind for 2 h at room temperature prior to washing, glycine elution, and amplification in *E. coli* XL-1 Blue cells (Table 1). In the third EDIII panning experiment, antigen from DENV1 and DENV2 was biotinylated with a 20 fold molar excess of NHS PEG4 Biotin reagent (Pierce) on ice for 2 h according to the manufacturers instructions (Thermo Fisher Scientific). The reaction was stopped with 100 mM glycine and excess biotin was removed by size exclusion chromatography. Both DENV1 and DENV2 were immobilized on streptavidin magnetic beads (Invitrogen) at a concentration of 100 nM for biopanning. Finally, when whole virus particles (DENV1) were used as bait, virus was coated in four wells of an immunoplate at 20 μg/mL in PBS, pH 7.4 prior to incubation with phage.

### 4.3. Construction and Biopanning of the Chimeric Fab-Phage Library

Total mRNA was prepared from 10 hybridomas that had been previously generated by immunizing BALB/c mice with EDIII from DENV2 TSV01 (AY037116), as described [20]. RT-PCR amplification of mouse Vκ and VH was performed for each hybridoma using published primers and protocols [25]. The Vκ and VH PCR products were pooled and mouse Vκ/human Cκ and mouse VH/human CH segments were assembled in overlap PCR reactions using 100 ng of each product with published protocols and primers [25]. A final overlap PCR was performed to combine the light and heavy chain segments into a single 1.5 kB product for cloning into the pComb3X with *Sfi*I as per published protocols [25].

The library was transformed into supercompetant *E. coli* XL-1 blue cells yielding approximately 2 × 10^6^ independent transformants. Selected clones from the library tranformation plate were sent for automated sequencing and screened for binding with DENV2 EDIII using a phage ELISA (method described below). The library was rescued with M13K07 helper phage and library phage (1 × 10^12^) was incubated with EDIII from DENV2 (10 μg/mL) coated in four wells of an immunoplate for biopanning. Washing, elution and amplification steps were performed as described above for the HX02 library.

### 4.4. Phage Clone Screening

Following the third (or final) round of selection individual *E. coli* clones were rescued with M13K07 and screened by ELISA for reactivity against EDIII or whole virus particles (coated in 50 mM Na_2_CO_3_ pH 9.6 or PBS pH 7.4, respectively). An anti-M13 horseradish peroxidase (HRP) conjugate (GE Healthcare) was used for detection and clones with an absorbance value two times higher than background levels were considered positive. Clone uniqueness was assessed with a *Bst*N1 restriction digest following PCR amplification of the Fab coding region of the phagemid as required. Clones with unique DNA fingerprints were subject to automated sequencing.

### 4.5. Expression and Purification of Fab and IgG

For HX02, phagemids from unique Fab-phage clones were digested with *Sal*I to remove the gene III coding sequence and re-ligated with T4 DNA ligase as described [34]. The pComb3X chimeric clones have an amber stop for direct expression in non-suppressor strains of *E. coli.* HX02 or pComb3X plasmids were transformed into *E. coli* Top10 F′ cells (Invitrogen) for expression and periplasmic extraction. Cell pellets were resuspended in chilled lysis buffer (120 mM Tris pH 8.0, 0.3 mM EDTA and 300 mM sucrose) and incubated on ice for 30 minutes for periplasmic extraction. Magnesium chloride (2.5 mM) was added to the clarified extract prior to immobilized metal affinity chromatography (IMAC) purification. Fabs were subject to a final purification step by SEC (S200 10/300 column) as required.

For expression of the C9 IgG the previously described PIGG vector was used [27], in which the heavy and light chains are expressed by an engineered bidirectional CMV promoter. The light chain encoding sequences of C9 were amplified using primers C9-light-5′ (GAGGAGAAGCTTGTTGCTC TGGATCTCTGGTGCCTACGGGGAACTCGACGTTTTGATG) with C-kappa 3′ (AATTATCTAG AATTAACACTCTCCCCTGTTGAAGCTCTTTGTGACGGGCAAGCTCAGGCCCTG) and cloned with *Hind*III/*Xba*I-ligation into PIGG. The heavy chain encoding sequences of C9 were amplified with primers C9-heavy-5′ (GAGGAGGAGCTCACTCCGAGGTTCAGCTTCAGCAATCTGG) and Universal heavy reverse (CCTGGCCGGCCTGGCCACTAGTGACCGATGGGCCCTTGGTGGAGGC) and cloned with ApaI/SacI-ligation into PIGG. The resulting plasmid was transfected into human embryonic kidney (HEK293T) cells with lipofectamine 2000 (Invitrogen, Carlsbad, USA) for transient IgG expression and subsequent purification on a recombinant Protein G column as previously described [34].

### 4.6. Elisa and Western Blot with Fab and IgG

For ELISA Maxisorb immunoplates were coated with 5 μg/mL EDIII in 50 mM Na_2_CO_3_ pH 9.6 or whole virus in PBS pH 7.4 and blocked with 5% skim milk in PBS-T. Blocked wells were incubated with purified Fab or IgG at room temperature for 1 h. Plates were washed with PBS-T and incubated with an anti-c-*myc* HRP conjugate (Roche) for detection of HX02 Fab or an anti-human HRP conjugate (MyBiosource) for detection of chimeric Fab and IgG.

For Western blot, infected cell extract, purified virus or EDIII proteins were separated by SDS-PAGE (12% or 15%), transferred to PVDF membrane, and Western blotted with Fab (100 nMol/L) or murine mAb 4G2 or 2H2 as controls. Anti-human or anti-mouse HRP conjugated secondary antibodies were used for detection. All blots were imaged on an ImageQuant RT ECL using ImageQuant TL software (GE Healthcare).

### 4.7. Affinity Maturation of C9 light Chain by Phage Display

The light chain CDR3 (LCDR3) library was constructed by saturation mutagenesis of the C9 Vκ CDR3 using published protocols [25,27]. The two fragments required for LCDR3 library assembly based on the C9 sequence were obtained with the PCR primer pairs OMPSEQ and F3-back (5′ GCAG AAATAAACTCCCAGATC) and C9-CDR3-NNK (5′ GATCTGGGAGTTTATTTCTGCNNKNNK GGTNNKNNKNNKNNKTGGNNKTTCGGTGGAGGGACAAAG) and GBACK. N is A, C, G or T and K is G or T. The bases encoding glycine (GGT) and tryptophan (TGG) that are within the CDR3, but were kept constant in the LCDR3 library synthesis, are underlined. Following an overlap PCR with the flanking primers (OMPSEQ and GBACK) the resulting 1.5 kB fragment with a randomized LCDR3 was cut with *Sfi*I and ligated into the appropriately digested pComb3X phagemid vector. The library was transformed into supercompetant *E. coli* XL-1 blue cells yielding approximately 4 × 10^5^ independent transformants. Twelve clones from the library transformation plate were subject to automated DNA sequencing and revealed different LCDR3 sequences.

The LCDR3 library was panned against DENV2 EDIII as described for the chimeric Fab-phage library with the inclusion of an “off-rate” election step [27]. In the second through the fourth cycles of panning 20 μg of DENV2 EDIII in PBS pH 7.4 was added to the well after removal of unbound phage by 5–10 washing steps. The plate was then incubated for 24 h at room temperature (off-rate selection) prior to five additional washing steps and glycine elution. After the fourth cycle, Fab-phage clones were tested for binding to immobilized EDIII by phage ELISA as described above.

### 4.8. DENV Immunostaining and Neutralization Assays

BHK-21 cells grown on cover slips at a density of 2 × 10^5^ were infected with DENV2 (GenBank accession EU081177.1) at an MOI of 0.3. Cells were fixed 24 h post-infection with 80% acetone at 4 °C for 15 min, and washed three times with PBS. Cells were blocked with 2% BSA in PBS overnight at 4 °C followed by an overnight incubation with primary antibody (C9 or 4G2, 13 nmol/L) at 4 °C. Cells were washed three times with PBS and incubated with goat anti-human or anti-mouse AF594 (Invitrogen, Carlsbad, USA) for 2 h at 4 °C. Labeled cells were washed three times with PBS, mounted with ProLong Gold containing DAPI (Invitrogen, Carlsbad, USA) and imaged using an inverted fluorescence microscope (Olympus IX71, Center Valley, USA) at 40× magnification. Image analysis was performed with ImageJ software [41].

Antibody neutralization was measured by a PRNT with BHK-21 cells as previously described [20]. Briefly, antibodies were pre-mixed with 50 pfu of DENV and incubated for 2 h at 4 °C prior to incubation with BHK-21 cells at 37 °C for 2 h. The mixture was replaced with RPMI 1640, 1% carboxymethylcellulose and antibody, and plates were incubated at 37 °C with 5% CO_2_ for 4–5 days. Plaques were manually counted following formaldehyde fixation.

## 5. Conclusions

The lack of EDIII specific Fab identified from a large naïve human library, together with a relative abundance of anti-DENV Fab in the same library, suggest EDIII antibodies are all but absent from the DENV-naïve human repertoire. Though previous phage display studies with WNV and B-cell immortalization from DENV infected donors suggest the EDIII response is amplified following infection, the levels of EDIII antibodies remain dramatically lower than those directed towards EDI and EDII [19,37]. This contrasts with studies in mice where DENV is non-pathogenic, unless animals are immune-compromised, and wild-type mice exhibit strong B-cell responses to EDIII [12,13,15]. It appears DENV, like other pathogenic viruses [42,43], has evolved its receptor-binding domain (EDIII) to have immunosilent properties in humans to facilitate cell entry.

## Figures and Tables

**Table 1:** Summary of phage display biopanning strategies against DENV envelope with the Naïve Human Fab Library (HX02).

Procedure	Strategy 1	Strategy 2	Strategy 3	Strategy 4
Antigen immobilisation	DENV1 EDIII Immunotube	DENV1 EDIII Immunoplate	Biotinylated EDIII (DENV1 and DENV2) Streptavidin resin	DENV1 whole virus Immunoplate
Antigen concentration	50 μg/mL (Pan1) 25 μg/mL, (Pan2&3)	10 μg/mL (Pan1–4) 1 μg/mL (Pan5&6)	100 nM (Pan1&2) 50 nM (Pan3–6)	20 μg/mL (Pan1&2) 10 μg/mL (Pan3&4)
Rounds of panning	Three rounds	Six rounds	Six rounds	Four rounds
*E. coli* strain	TG1	XL-1 Blue	TG1	XL-1 Blue
Clone screening	190 (Pan3)	95 (Pan4), 95 (Pan5), 95 (Pan6)	95 (Pan3), 285 (Pan5), 190 (Pan6)	384 (Pan4)
DENV positive clones	Nil	Nil	Nil	188/384
